# In vitro characterisation of low-cost synthetic meshes intended for hernia repair in the UK

**DOI:** 10.1007/s10029-021-02401-z

**Published:** 2021-04-02

**Authors:** A. Grillo, Z. Hyder, V. Mudera, A. Kureshi

**Affiliations:** 1 Centre for 3D Models of Health and Disease, Division of Surgery and Interventional Science, University College London, London, UK; 2 Hydermed Limited, London, UK

**Keywords:** Hernia repair, Low-cost mesh, Biocompatibility, Mechanical tests

## Abstract

**Purpose:**

Low-cost meshes (LCM) were repurposed for the repair of hernias in the developing world. In vivo studies have shown LCM to have comparable results to commercial meshes (CM) at a fraction of the cost. However, little has been done to characterise the mechanical and biocompatible properties of LCM, preventing its clinical use in the UK. The objectives of the research are to assess mechanical and ultrastructural properties of two UK-sourced low-cost meshes (LCM) and the characterisation of the LCMs in vitro biocompatibility.

**Methods:**

Mechanical properties of the two LCM were measured through uniaxial tensile test and ultrastructure was evaluated with Scanning Electron Microscopy. LIVE/DEAD^®^ Viability/Cytotoxicity Assay kit and alamarBlue were used to assess cellular viability and proliferation, respectively. Images were acquired with a fluorescence microscope and analysed using ImageJ (NIH, USA).

**Results:**

LCM1 and LCM2 were both multifilament meshes, with the first having smaller pores than the latter. LCM1 exhibited significantly higher tensile strength (*p* < 0.05) than LCM2 but significantly lower extensibility (*p* < 0.0001), while Young’s Modulus of the two samples was not significantly different. No significant difference was found in the cellular viability and morphology cultured in LCM1 and LCM2 conditioned media. Metabolic assay and fluorescence imaging showed cellular attachment and proliferation on both LCMs over 14 days.

**Conclusion:**

The characterisation of the two UK-sourced LCMs showed in vitro biocompatibility and mechanical and ultrastructural properties comparable to the equivalent CM. This in vitro data represents a step forward for the feasibility of adopting LCM for surgical repair of hernias in the UK.

## Introduction

In the developing world, hernias represent a serious burden for the local healthcare systems. The reasons for this are the high prevalence and severity of the condition mainly due to the numerous neglected cases and the scarce resources available for surgical treatment [1]. However, the use of a low-cost material, typically used as mosquito netting, has been reported to repair the hernia defect and showed comparable outcomes to the more expensive commercial meshes (CM), importantly at a fraction of the cost [2–4]. The regulatory pathway and safety validations needed before the commercialisation of a product surely contributes to the high cost of CM. Nonetheless, evidence from various studies suggests that this frugal approach has great potential to be utilised here in the UK, saving the NHS millions of pounds, if appropriately scrutinized under UK regulations to ensure safety [5]. However, a lack of their characterisation along with other barriers, such as appropriate sterilisation to satisfy regulations in developed countries, has prevented its widespread use.

CM meshes are usually made of synthetic materials such as polypropylene, polyester or nylon. Similarly, low-cost meshes (LCM) are also made of these different materials and have low material content which classifies them as lightweight. The weight of a mesh depends on the amount of material used and determines the intensity of the foreign body reaction and scarring: the higher the material content, the more severe the immune response triggered in the body, making lightweight meshes favourable for implantation [6]. Also, pore size influences the success of a mesh after implantation. It is suggested that the pore size should be large enough (> 800 µm) to allow infiltration of fibroblasts and macrophages to avoid infections and promote regeneration of the tissue. Large pores also avoid the formation of granuloma bridging which leads to a stiff scar plate and therefore a reduction in flexibility of the tissue [6, 7]. Previous studies on LCM have demonstrated they usually have pore sizes larger than 1 mm, which would be advantageous for the above reasons [8, 9].

Other features usually evaluated in CM include mechanical properties. Since the function of the mesh in the hernia repair is to reinforce the abdominal wall, it is important for the mesh to have comparable properties to those of the native tissue. However, commonly adopted CM usually has a greater tensile strength than the fascial tissue that they are going to reinforce, producing a mismatch of properties [10]. It is thought that this mechanical discrepancy may be the cause of postoperative complications such as recurrence, pain and prolapse [11].

Considering the biocompatibility of LCM, most studies examined the effects of LCM directly in vivo (either animal or human) showing the success of LCM [2–4, 12, 13]. The concern of performing exclusively in vivo studies resides in the fact that there is a huge variation in this type of experiment as different animal species are used (rabbit, pig, rat) and therefore comparison is difficult. While an initial in vitro assessment would give more consistent and comparable results it would also reduce the number of in vivo experiments. However, limited in vitro biocompatibility assessments of LCM and cell attachment are published. Sanders, Kingsnorth [14] demonstrated that bacteria adherence on LCM compared to CM with no significant difference between the two types. Also, Wiessner, Kleber [15] tested the in vitro toxicity of LCM compared with CM through culture with fibroblasts but visual evidence of cells attaching and proliferating on the mesh is lacking. To our knowledge, there is no published research which performs a full characterisation of LCM analysing structure, mechanics and in vitro biocompatibility and attachment of cells to LCM.

The aim of this study is to characterise the mechanical and ultrastructural properties of two low-cost meshes sourced from the UK. Their biocompatibility is also assessed, focusing on cell morphology, viability, attachment, and proliferation. Importantly, this will be a vital step towards assessing the feasibility of utilising LCM for hernia repair and reduce the skepticism around the use in the UK.

## Materials and methods

Two low-cost meshes termed LCM1 and LCM2, fabricated from nylon (Mountain Warehouse, UK) and polyester (Purple Turtle, UK) respectively, were investigated. Pore size was calculated with the imaging software ImageJ as the longest distance between two edges of a pore. Weight was calculated as weight of the mesh per area (mm^2^).

Human Dermal Fibroblasts (HDFs) were cultured in high glucose DMEM (Sigma Aldrich, UK) supplemented with 10% FBS (Gibco, Thermo Fisher Scientific) and 1% Penicillin/Streptomycin (Thermo Fisher Scientific) (passage number 7) HDFs were sub-cultured using Trypsin – EDTA (Thermo Fisher Scientific) when at 80–90% confluence.

### Ultrastructure

To analyse LCM ultrastructure, samples of the meshes were dehydrated using ethyl alcohol and were mounted on aluminium pin stubs and sputter-coated with gold/palladium (Polaron E5000, Quorum Technology, UK) prior to being examined with a Scanning Electron Microscope FEI XL30 FEGSEM (FEI UK, UK) operating at 5 kV.

### Mechanical testing

To perform mechanical tests, samples of nylon mesh were cut into dog-bone shapes (width: 10 mm; grip-to-grip distance—20 mm) and a uniaxial tensile test system was used (BT1-FR5.0TN, Zwick Roell Group, Ulm, Germany). The system was attached to a 0.5 kN loading cell (KAP-TC, Zwick Roell Group, Ulm, Germany) which measures the force applied to the sample through the grip and the test was conducted in displacement-controlled mode (8 mm/min). The thickness of the samples was measured using a thickness gauge (Mitutuyo 543-402BS, Sakado, Japan) with a resolution of 0.01 mm. Samples that did not break at their centre were not included in the dataset. Break stress (strength) was calculated as the maximum force divided by the grip-to-grip distance (N/cm). Break strain was measured as the value of strain at maximum load, as percentage (%) of extension. Young’s Modulus (stiffness) was measured considering the gradient of the stress–strain (force-extension) curve and selecting the slope of the linear region (N/cm).

### Biocompatibility

#### Cytotoxicity and morphology

Cytotoxicity was evaluated according to the ISO 10993-5 standard. Human adult-donor Dermal Fibroblasts (HDF) were kindly gifted by Prof. Umber Cheema and originally purchased through Promocell (Heidelberg, Germany). HDFs (passage number 7) (10,000 cells/well in 24-well plate) were grown in mesh-conditioned media. This was prepared by sterilising 300 cm^2^ of each mesh with 70% ethanol and Phosphate Buffer Solution (PBS) prior to immersing it in 50 ml of DMEM supplemented with FBS and Penicillin/Streptomycin for 24 h at 37 °C, 5% CO_2_.

Cell viability was assessed with a LIVE/DEAD^®^ Viability/Cytotoxicity Assay kit (Thermo Fisher Scientific—Life Technologies). Control samples for cell viability and morphology were represented by culture in DMEM supplemented with FBS and P/S (CTRL−) and 70% methanol (CTRL +) for viability tests. Cell morphology was evaluated following fixation of samples in 4% paraformaldehyde for 30 min and washed with Phosphate Buffer Solution (Thermo Fisher Scientific). After permeabilization using 0.25% Triton X-100 (Sigma Aldrich), samples were washed in PBS and stained using Phalloidin FITC (Sigma) 1:1000 in PBS for 1 h to visualise cell’s actin filaments. Following these steps, the samples were mounted on glass microscope slides with VECTASHIELD® DAPI (Vector Laboratories Inc.) to visualise nuclei. Samples were imaged with a Zeiss inverted microscope and analysed using the image processing program ImageJ (NIH, USA).

#### Cell attachment and proliferation

Biocompatibility and cell attachment to the mesh were evaluated by seeding HDFs (10,000 cells/well) onto ethanol-sterilised mesh samples (15.6 mm diameter). The seeding efficiency of LCM1 and LCM2 was 10 and 5%, respectively (data not shown). Samples were analysed at 3, 7, 14 days.

Proliferation of HDFs seeded on the meshes was evaluated with alamarBlue (Invitrogen, Thermo Fisher), with 1:9 ratio (alamarBlue to growing media ratio). Control samples were HDFs cultured in DMEM supplemented with FBS and P/S. Samples were incubated for 4 h at 37 °C, 5% CO_2_ and transferred to a 96-well plate (triplicates of 100 µl from each sample). Samples were imaged with a CLARIOstar^®^ microplate reader (BMG LABTECH GmbH, Germany) in fluorescence mode (excitation 560 nm, emission 590 nm).

Samples of the seeded meshes were fixated in 4% paraformaldehyde for 30 min and washed with Phosphate Buffer Solution (Thermo Fisher Scientific). After permeabilization using 0.25% Triton X-100 (Sigma Aldrich), samples were washed in PBS and stained using Phalloidin TRITC (Sigma) 1:1000 in PBS for 1 h to visualise cells’ actin filaments. Following these steps, the samples were mounted on glass microscope slides with VECTASHIELD^®^ DAPI (Vector Laboratories Inc.) to visualise nuclei. Samples were imaged with Zeiss AxioObserver Microscope with ApoTome.2 feature and Zeiss ZEN software (Zeiss, Oberkochen, Germany).

## Results

### Ultrastructure characterisation

**Fig. 1:**
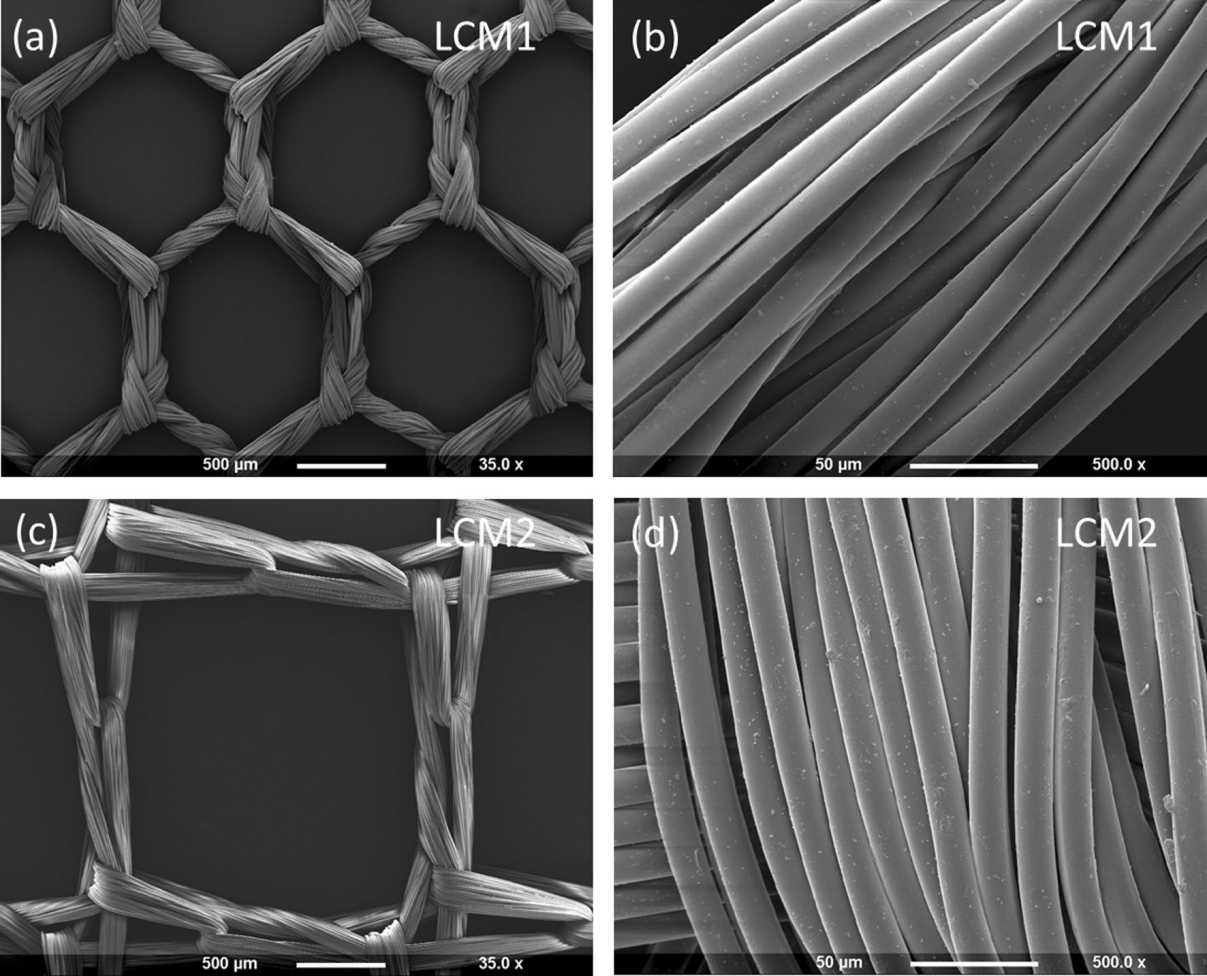
Micrographs showing LCM1 and LCM2 at 35× (**a**, **c**) and 250× (**b**, **d**) obtained with Scanning Electron Microscope. Figures (**a**) and (**c**) show low magnification images (35×) of the meshes which allow to determine their pore size and shape. Figures (**b**) and (**d**) are high magnification images (500×) showing the multifilament arrangement of the fibres composing the mesh

Ultrastructural characterisation was performed to evaluate LCM parameters and compare them to existing data on low-cost meshes and commercial meshes. SEM analysis showed that both LCM1 and LCM2 are multifilament meshes, as shown in Fig. 1b, d where a magnification of a single fibre of the mesh reveals its multifilament organisation. These filaments have almost identical diameters of approximately 14 µm (LCM1 is 14.95 ± 1.45 µm; LCM2 is 14.76 ± 1.59 µm), showing no statistical difference between the two (Table 1).

**Table 1:** Table showing structural parameters of LCM1 and LCM2. All measurements were obtained from three samples of each mesh type

	Pore size (mm)	Pore area (mm^2^)	Weight (g/m^2^)	Pore density (pores/cm^2^)	Thickness (mm)	Filament diameter (µm)
LCM1	1.16 ± 0.07	0.79 ± 0.03	34.3	73	0.16	14.95 ± 1.45
LCM2	2.25 ± 0.05	2.16 ± 0.18	29.3	21	0.16	14.76 ± 1.59

Figure 1 shows that the two meshes differ in pore shape, where LCM1 has hexagonal pores (Fig. 1a) and LCM2 has square pores (Fig. 1c).

Pore sizes of the two meshes are specified in Table 1. LCM2 has a larger pore size than LCM1, 2.25 ± 0.05 mm and 1.16 ± 0.07 mm, respectively. Despite a difference in pore size for the two meshes, according to a standard proposed by Earle and Mark [16], they are both considered large pores since their pore diameter is larger than 1 mm.

Another important feature for the characterisation of meshes is weight, considered as the amount of material per area of the mesh (g/m^2^). Considering the two meshes, LCM2 (29.3 g/m^2^) has a lower value of weight than LCM1 (34.3 g/m^2^). According to the classification introduced by Earle and Mark [16] both meshes are identified as lightweight.

### Mechanical characterisation

**Fig. 2:**
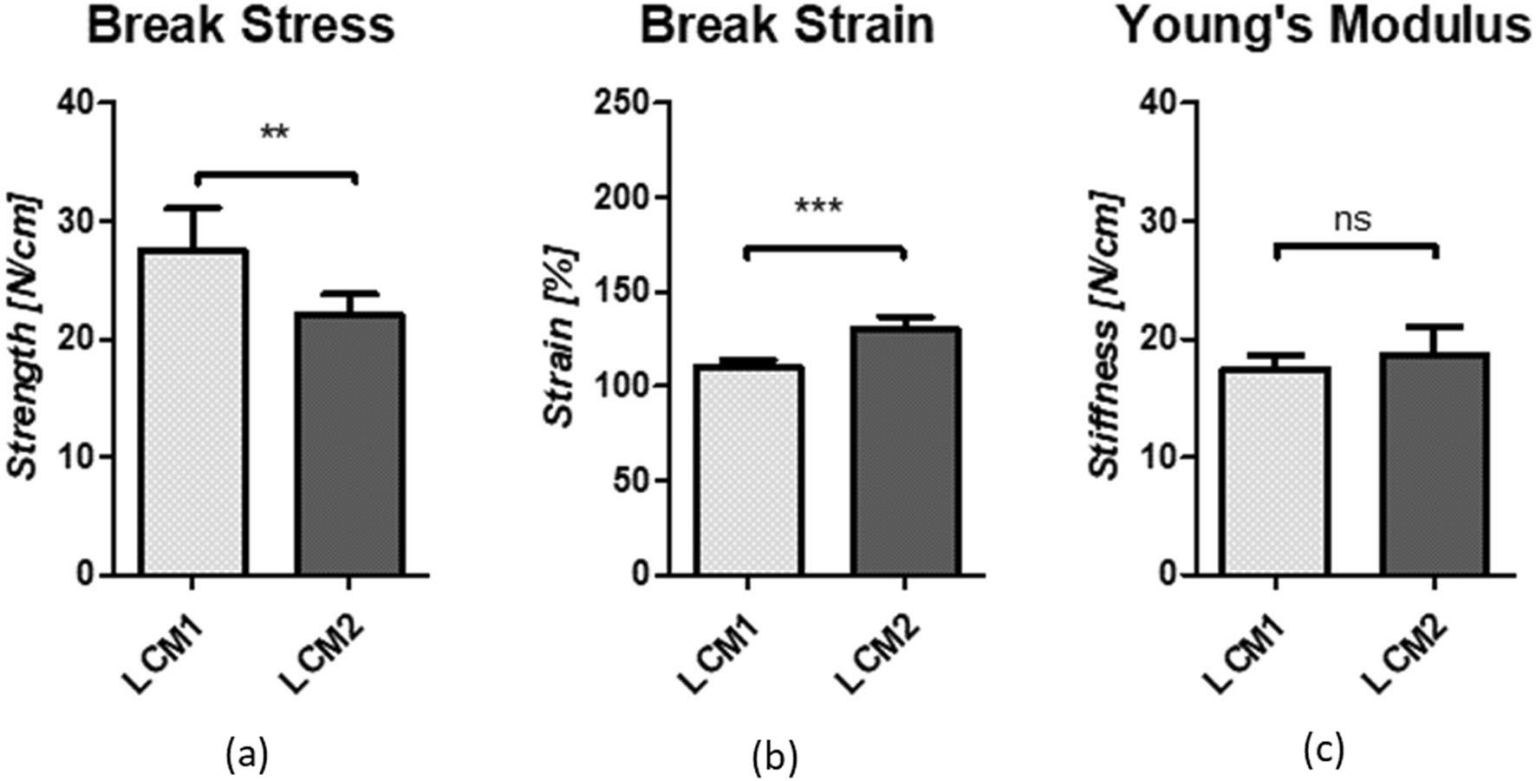
Graphs showing break stress (**a**), break strain (**b**) and Young’s Modulus (**c**) of LCM1 and LCM2 obtained with uniaxial tensile test. Values are expressed as mean ± SEM, *N* = 8. Data were analysed using unpaired *t* test. **p < 0.05; ***p < 0.0001; *ns* non-significant

Uniaxial tensile tests were performed on the two low-cost meshes to define their mechanical properties: strength (break stress), extensibility (break strain) and stiffness (Young’s Modulus). Eight samples per mesh were tested until failure, indicated by the specimen breaking at the middle point. Mean of the break stress, break strain and Young’s Modulus of LCM1 and LCM2 were compared, and their statistical significance was assessed with an unpaired t-test. Mechanical characterization revealed that LCM1 had a significantly higher tensile strength than LCM2, 27.5 ± 3.5 and 22.0 ± 1.7 N/cm, respectively (*p* < 0.05) (Fig. 2a). On the contrary, LCM1 had significantly lower extensibility than LCM2, with values of 110.3 ± 3.4% for LCM1 and 130.5 ± 6.3% for LCM2 (*p* < 0.0001) (Fig. 2b). Young’s Modulus, which represents the stiffness of the material, was calculated from the slope in the linear region of the force–extension graph. The stiffness measurements were very similar for both LCM2 (17.4 ± 1.2 N/cm) and LCM1 (18.7 ± 2.3 N/cm). Both meshes fall in the definition of large pores for meshes and this may explain the fact that the stiffnesses of the two meshes are similar. LCM2 has a significantly higher break strain (*p* < 0.0001) which could be explained by the different pore geometry which may influence the extension of the material, causing altered deformation of the mesh when undergoing a tensile test.

### Cytotoxicity and morphology

**Fig. 3:**
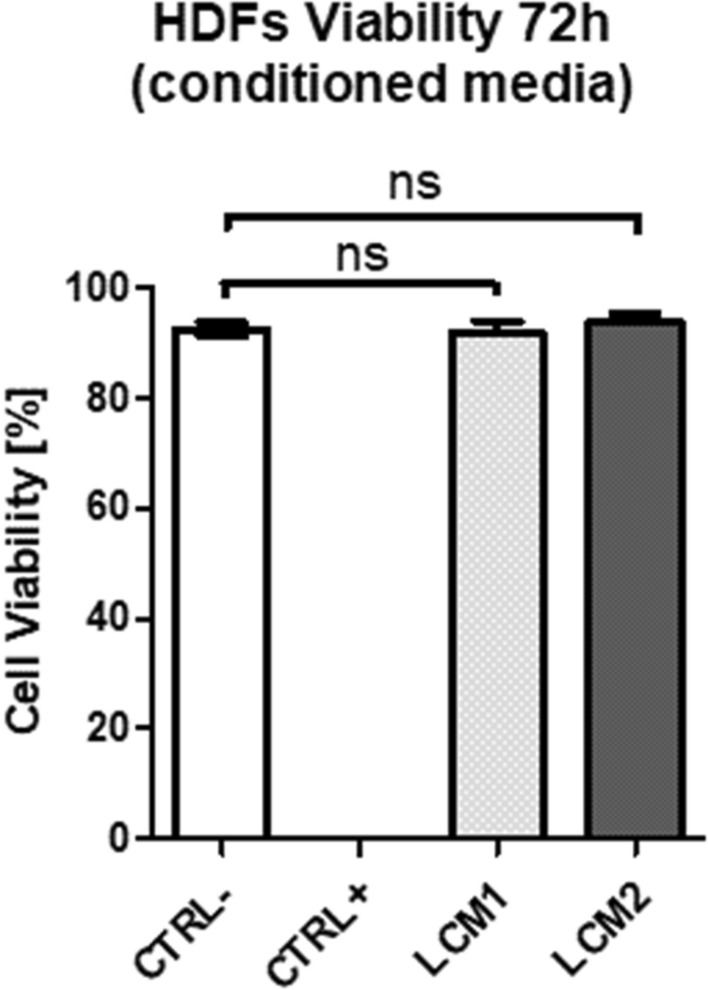
Graph showing HDFs viability after 72 h in mesh-conditioned media. Quantitative analysis of HDFs viability after 72 h in mesh-conditioned media using LIVE/DEAD assay. CTRL− represents viability in DMEM, CTRL + in 70% methanol, LCM1 and LCM2 in large and small pore conditioned media, respectively. Values are expressed as mean ± SEM, *N* = 3. Data were analysed using the unpaired *t* test between the control and the two conditions (LCM1 and LCM2)

**Fig. 4:**
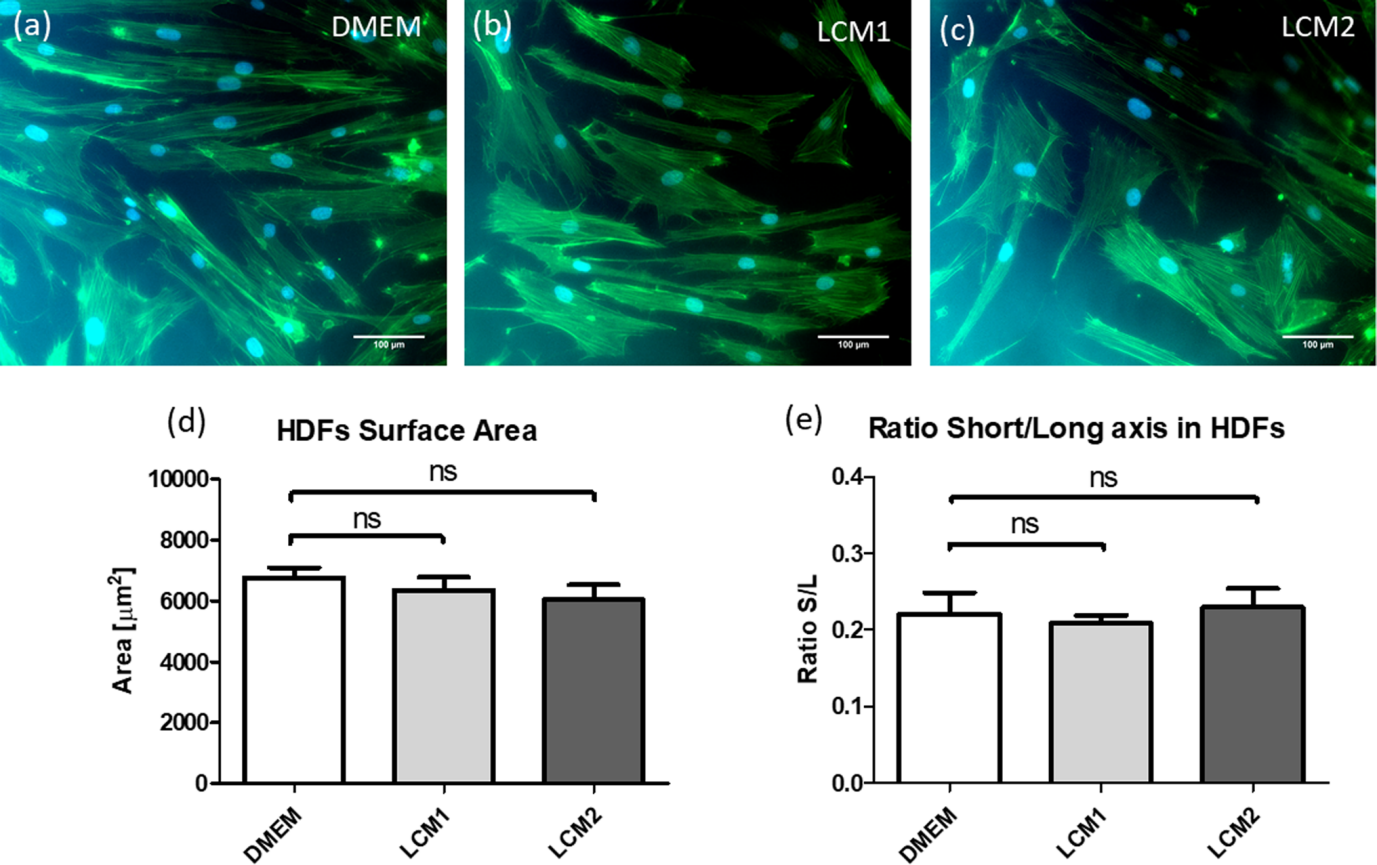
Figures showing HDFs morphology after 72 h in LCM1 and LCM2 conditioned media and DMEM (control). Scale bar in the micrographs is 100 µm. HDFs were grown in mesh-conditioned media and DMEM for 3 days. Graphs show quantitative analysis of morphological parameters of HDFs. Graph (**d**) represents the surface area of the cells calculated in the frame of the image; graph (**e**) represents the ratio between the short and long axis of the cell. In both morphological analysis five images were analysed and cells counted in the frame and the control group (DMEM) was compared with mesh conditioned media groups (LCM1 and LCM2). Values are expressed as mean ± SEM, *N* = 5. Data were analysed using the unpaired *t* test between the control and the two conditions. Images were analysed using ImageJ

Cytotoxicity tests were performed to determine if LCM1 and LCM2 released toxic components that could adversely affect cell viability and proliferation. HDFs were cultured in mesh-conditioned media obtained from LCM1 and LCM2. Figure 3 shows that the percentage of viable cells in DMEM is not significantly different from that of LCM1 or LCM2 conditioned media, confirming that cell viability is not compromised by the two meshes. Morphology of HDFs cultured in DMEM and mesh-conditioned media was qualitatively and quantitatively evaluated. Figure 4 shows immunofluorescence images of HDFs cultured in DMEM and mesh-conditioned media for 72 h. No difference in morphology of HDFs between test and control conditions was observed. This was confirmed by the quantitative analysis carried out on the images. Briefly, cellular surface area and ratio between the short and long axis were measured from all the conditions using imaging software ImageJ. Figure 4d shows the surface area of HDFs cultured in DMEM and the two conditioned media samples and no significant difference is seen between the two groups. Figure 4e illustrates that there is no significant difference in HDF morphology between the control and the LCM1 and LCM2 conditioned media.

### Cell attachment and proliferation

**Fig. 5:**
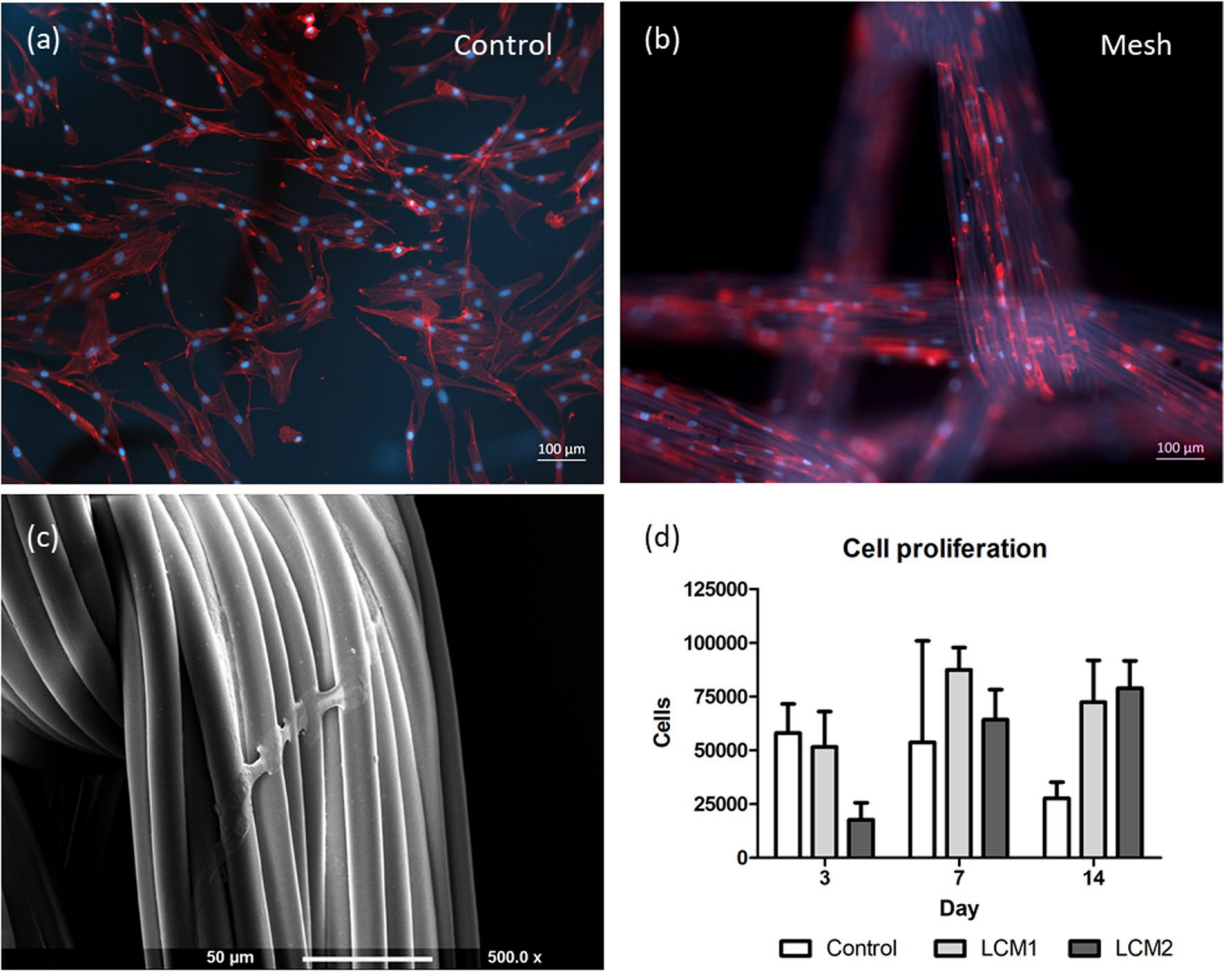
Representative immunofluorescence (**a**, **b**) and SEM (**c**) images and proliferation activity (**d**) of HDFs attached on plastic (control), LCM1 and LCM2. HDFs (10,000 cells/sample) were seeded on plastic, as control, (**a**) and meshes (**b**) and cultured for 14 days. Seeding density was derived by calculating the seeding efficiency on the meshes (5 and 10% on LCM1 and LCM2, respectively). Immunofluorescence images show HDFs stained with DAPI (blue) and phalloidin (red) for visualizing nuclei and actin filaments, respectively (scale bar 100 µm). SEM micrograph (**c**) shows a cell attached across the filaments of a fibre in the LCM2 mesh at day 3 (scale bar 50 µm). Cellular proliferation (**d**) of HDFs on plastic and LCM1 and LCM2 was evaluated using alamarBlue assay at day 3, 7, 14, *N* = 3 to show proliferation of HDFs on the meshes. Values are expressed as mean ± SD, *N* = 3

Cellular attachment and proliferation of HDFs were tested to evaluate if meshes represented a suitable environment for the maintenance/growth of cells. HDFs were seeded on LCM1 and LCM2 and compared with HDFs seeded on a plastic well. Initial seeding density (10,000 cells/ml) was determined after evaluating the seeding efficiency of HDFs on both meshes [this was 5 and 10% for LCM1 and LCM2, respectively (data not shown)]. Figure 5a, b show representative images from immunofluorescence staining of HDFs cultured on plastic and LCM2, respectively, at day 3. HDFs successfully attached to the mesh and proliferated along the mesh filaments. Morphology of HDFs on the 3D structure of the fibres shows no difference with those grown in the plastic well on a 2D surface, as they exhibit an elongated shape on both surfaces. Figure 5c is a scanning electron microscopy image showing cellular attachment across the filaments of the fibre instead of along it, as it was visible in the immunofluorescence images.

To verify the qualitative observations, proliferation of HDFs seeded on meshes and plastic was evaluated at three time points (3, 7, 14 days) to calculate cell number and therefore the proliferation curve. The proliferation data were inferred from the metabolic activity of the HDFs seeded on the mesh samples and in the well at the specific time points using a standard curve produced using ascending cell densities (2.5 × 10^4^–12.5 × 10^4^ cells/ml) (*R*^2^ = 0.9944) (data not shown). Figure 5d was plotted according to the standard curve previously described and shows the proliferations of HDFs on plastic (control), LCM1 and LCM2 over 14-week period. HDFs seeded on LCM2 proliferated from almost 1.8 × 10^4^ cells (1.8 × 10^4^ ± 0.8 × 10^4^ cells) at 3 days to almost 8.0 × 10^4^ cells (8.0 × 10^4^ ± 1.3 × 10^4^ cells) at 14 days, in an ascending curve. At 3 days, HDFs on LCM1 and the control are just above 5.1 × 10^4^ (5.2 × 10^4^ ± 1.7 × 10^4^ cells) and 5.8 × 10^4^ (5.8 × 10^4^ ± 1.3 × 10^4^ cells) respectively; at 7 days there is an increase of HDFs on LCM2 (8.8 × 10^4^ ± 1.0 × 10^4^ cells) while for the control there is a decrease to almost 5.4 × 10^4^ cells (5.4 × 10^4^ ± 4.7 × 10^4^ cells). By 14 days, cell proliferation of the control had decreased to almost 3.0 × 10^4^ cells (2.7 × 10^4^ ± 0.7 × 10^4^ cells) while both LCM1 and LCM2 had an overall increase in cell proliferation from day 3 with a cell count between 7.2 × 10^4^ and 7.8 × 10^4^ (7.2 × 10^4^ ± 1.9 × 10^4^ and 7.9 × 10^4^ ± 1.3 × 10^4^ cells, respectively). The different trend exhibited by the control compared with LCM1 and LCM2 could be explained considering that HDFs reached a higher confluence on the well than on the mesh. In fact, the surface of the well is a 2D structure and therefore possesses less area for cells to attach. In contrast, meshes (LCM1 and LCM2 in the specific experiment) are a 3D structure and possess a higher surface area for cells to grow. Figure 5b shows how cells attach on top and in between filaments of the mesh.

## Discussion

Low-cost mesh is being used in the developing world as a frugal alternative to commercial medical grade mesh for hernia repair. Importantly, it was previously demonstrated to provide similar surgical outcomes to expensive CM at a fraction of the cost [1–4]. Despite this huge advantage, it is yet to be used in the developed world and as such, has the potential to introduce substantial saving for healthcare systems. However, there are many barriers to its adoption here in the UK. The huge variation in types of materials and lack of pre-clinical characterisation are just some contributing factors. This study has demonstrated characterisation of the mechanical, structural and biocompatibility of two LCM available for purchase here in the UK. Our results indicate that these LCM are comparable to other LCM being utilised in the developing world since they are lightweight and with large pores. The two meshes investigated in this study (LCM1 and LCM2) have a pore size ranging between 1 and 2.2 mm similar to the other low-cost meshes used in the developing world, and therefore considered large pore [16]. This is an important feature since it was demonstrated that the pore size should be large enough to allow macrophages, fibroblasts, and blood vessels to infiltrate, trigger tissue in-growth and avoid the arise of infections [6, 7]. Considering the weight, the LCM analysed in this study and those used in Ghana, Burkina Faso and Cameroon are considered lightweight according to the classification proposed by Kalaba, Gerhard [17], which makes them a favourable choice for implantation. In fact, recent studies have confirmed that lightweight meshes induce a less pronounced foreign body reaction compared to heavyweight meshes because of the high content of material [6]. The two low-cost meshes used in this study have a weight of 34.3 and 29.3 g/m^2^ and are classified as lightweight, which makes them similar to the low-cost meshes already used in other studies and to the lightweight commercial meshes. They also have mechanical properties more similar to native tissue than over-engineered CM which, with their higher stiffness, give rise to complications such as poor integration and pain.

Mechanical properties are important features to consider when designing or choosing a mesh for hernia repair. Historically, the first meshes were designed to have high strength and high material content. However, later studies demonstrated that those over-engineered meshes led to complications such as stiffening of the mesh after implantation and restriction of movement for the patient because of the evident mismatch of properties with the abdominal wall. Conversely, the use of meshes with similar mechanical properties to the native tissue and lower material content reduced these complications achieving better integration [6]. Low-cost meshes currently used for inguinal hernia repair are fabricated from a range of materials (polypropylene, polyester, nylon) and exhibit differences in mechanical strength, usually comparable to lightweight CM [9]. Likewise, CM usually present great variability in their mechanical properties, materials, and structure. In fact, CM can present tensile strength ranging from 10 to 100 N/cm, pore size from few micrometres to 3 mm and a large variability of materials (polypropylene, polyester, nylon) and coatings [6, 18]. However, even for the CM the ideal mesh does not exist and Brown and Finch [6] suggest that the surgeon should consider the conditions of the repair to be performed when choosing a mesh, even though usually lightweight meshes with large pores and tensile strength similar to the native tissue are preferable. For this reason, a more thorough analysis of LCM would help in their acceptance in clinical applications. Moreover, the low-cost meshes used in poorer settings are usually implanted without any type of pre-clinical characterisation and this lack of information makes it impossible to correlate mesh properties with their success or failure.

An initial objective of our study was to determine the mechanical strength, extensibility, and stiffness (break stress, break strain, and Young’s modulus) of two low-cost meshes purchased in the UK. Their values of break stress were compared with those found in the study by Ambroziak, Szepietowska [19] where four polyester low-cost meshes were tested for tensile strength and strain. Their results showed that the tensile strength ranged between 12.8 and 23.2 N/cm, which are comparable to the values found in the present study where the values of break stress were 27.5 ± 3.5 and 22.0 ± 1.74 N/cm for LCM1 and LCM2, respectively. Another example of low-cost meshes successfully used for hernia repair is represented by the polyester mosquito mesh implanted in the study of Rouet, Bwelle [20] which had a tensile stress of 20 N/cm. Furthermore, Sanders, Kingsnorth [9] compared the polyethylene mosquito mesh which was used by the non-profit organization “Operation Hernia” with some common commercial meshes. It was found that its pore size was identifiable as large pore and the tensile strength (42.7 and 31.5 N/cm for vertical and horizontal direction, respectively) was higher than the low-cost meshes considered in the present study but considerably lower than the commercial meshes analysed. These previous studies analysing the mechanical properties of LCM sourced in the developing world, found that tensile strength is comparable with that of most common lightweight meshes CM and therefore more similar to that of abdominal wall tissues than heavyweight CM. However, the lack of standardisation in expressing mechanical properties for meshes complicates the comparison of low-cost meshes among different studies. For example, some studies express the load at which the mesh breaks, in Newton (N), while others indicate the tensile strength, in Newton per centimetre (N/cm) and some others express the tensile strength in Pascals, considering the load over the cross-section of the sample. However, as the initial size of the sample is often not specified in the methods, it is not possible to convert the measures from one unit to the other and therefore draw comparisons to different studies.

In the process of characterising LCM, the assessment of the material type is an important step to clarify its properties and possible reactions after implantation in the body. This involves the assessment of the material’s biocompatibility. Low-cost meshes are usually used in emergency situations where commercial meshes are not available due to their high cost. For these reasons, there are many studies involving implantation and study of low-cost meshes directly in animals and in humans. However, studies involving in vivo experiments use different animal models (rat, pig, rabbit), and the variability between species makes it difficult to draw comparisons and correlate to human hernia repair.

Although animal models are an essential step in the pathway to clinical applications, performing in vitro experiments prior to in vivo studies, would provide useful information about the materials and help to better predict mesh response, which for CM is currently lacking [21]. This approach would help optimise the design of further in vivo experiments, enabling a reduction in animal testing. Among the few studies which analyse in vitro biocompatibility of meshes, Sanders, Kingsnorth [14] examined the in vitro adherence of bacteria on commercial meshes compared to low-cost meshes and found no significant difference between them, confirming their suitable sterility for human implantation. Wiessner, Kleber [15] showed the biocompatibility of CM and sterilised LCM through culture with fibroblasts. In their analysis, it was demonstrated that there were no significant differences in biocompatibility between commercial and low-cost meshes although no visual evidence was given of fibroblasts attaching and proliferating on the meshes. On the contrary, our study showed how fibroblasts attached and proliferated on both low-cost meshes, demonstrating the biocompatibility of UK sourced LCM.

Numerous studies have demonstrated the safety and success of low-cost meshes for hernia repair. Nonetheless, further steps are still required for their acceptance worldwide and appropriate sterilisation is a key issue preventing their use. In fact, in most studies LCM were implanted following steam sterilisation at 121 ˚C for 15 min. This approach was proven to be effective at preventing infections after implantation and cost-effective compared to the more expensive techniques commonly used for CM, such as Ethylene Oxide sterilisation [9, 22]. However, UK guidelines recommend sterilisation at 134 ˚C for 3 min for medical devices which in most cases is not applicable to LCM because the composition of these materials is unable to withstand such high temperatures without undergoing structural and mechanical alterations [8, 22, 23]. A future step to promote the use of MM in developed countries could involve the comparison of the effects of different sterilising temperatures on LCM1 and LCM2 and their subsequent changes in structure, biocompatibility and mechanical properties.

In conclusion, this study is the first to present the characterization of UK-sourced low-cost meshes including mechanical properties, ultrastructure and biocompatibility. According to the results obtained in this study, LCM2 displayed favourable features of larger pore size and lower weight; important characteristics needed for optimal integration in the body and a low inflammatory response. Moreover, LCM2 showed a lower tensile strength and similar stiffness to LCM1 but both lower than common CM. An important observation from this study is the assessment of biocompatibility, in particular for LCM2 for the attachment and proliferation of HDFs on the mesh. Despite the numerous measures still remaining to establish the safety and adoption of this frugal innovation worldwide, this research demonstrates the potential benefits of UK-sourced LCM over CM and takes us a step towards its implementation for hernia repair in the UK.
